# The P_II_-NAGK-PipX-NtcA Regulatory Axis of Cyanobacteria: A Tale of Changing Partners, Allosteric Effectors and Non-covalent Interactions

**DOI:** 10.3389/fmolb.2018.00091

**Published:** 2018-11-13

**Authors:** Alicia Forcada-Nadal, José Luis Llácer, Asunción Contreras, Clara Marco-Marín, Vicente Rubio

**Affiliations:** ^1^Instituto de Biomedicina de Valencia del Consejo Superior de Investigaciones Científicas, Valencia, Spain; ^2^Departamento de Fisiología, Genética y Microbiología, Universidad de Alicante, Alicante, Spain; ^3^Group 739, Centro de Investigación Biomédica en Red de Enfermedades Raras – Instituto de Salud Carlos III, Valencia, Spain

**Keywords:** protein structure, nitrogen regulation, gene expression regulation, signaling, P_II_ complexes, PipX complexes, NtcA structure and complexes, PlmA

## Abstract

P_II_, a homotrimeric very ancient and highly widespread (bacteria, archaea, plants) key sensor-transducer protein, conveys signals of abundance or poorness of carbon, energy and usable nitrogen, converting these signals into changes in the activities of channels, enzymes, or of gene expression. P_II_ sensing is mediated by the P_II_ allosteric effectors ATP, ADP (and, in some organisms, AMP), 2-oxoglutarate (2OG; it reflects carbon abundance and nitrogen scarcity) and, in many plants, L-glutamine. Cyanobacteria have been crucial for clarification of the structural bases of P_II_ function and regulation. They are the subject of this review because the information gathered on them provides an overall structure-based view of a P_II_ regulatory network. Studies on these organisms yielded a first structure of a P_II_ complex with an enzyme, (N-acetyl-Lglutamate kinase, NAGK), deciphering how P_II_ can cause enzyme activation, and how it promotes nitrogen stockpiling as arginine in cyanobacteria and plants. They have also revealed the first clear-cut mechanism by which P_II_ can control gene expression. A small adaptor protein, PipX, is sequestered by P_II_ when nitrogen is abundant and is released when is scarce, swapping partner by binding to the 2OG-activated transcriptional regulator NtcA, co-activating it. The structures of P_II_-NAGK, P_II_-PipX, PipX alone, of NtcA in inactive and 2OG-activated forms and as NtcA-2OG-PipX complex, explain structurally P_II_ regulatory functions and reveal the changing shapes and interactions of the T-loops of P_II_ depending on the partner and on the allosteric effectors bound to P_II_. Cyanobacterial studies have also revealed that in the P_II_-PipX complex PipX binds an additional transcriptional factor, PlmA, thus possibly expanding PipX roles beyond NtcA-dependency. Further exploration of these roles has revealed a functional interaction of PipX with PipY, a pyridoxal-phosphate (PLP) protein involved in PLP homeostasis whose mutations in the human ortholog cause epilepsy. Knowledge of cellular levels of the different components of this P_II_-PipX regulatory network and of K_D_ values for some of the complexes provides the basic background for gross modeling of the system at high and low nitrogen abundance. The cyanobacterial network can guide searches for analogous components in other organisms, particularly of PipX functional analogs.

Protein P_II_ was discovered in the late sixties of last century (Stadtman, 2001), when *Escherichia coli* glutamine synthetase (GS) was found to exist in feed-back inhibition susceptible or refractory forms depending on the adenylylation state of one tyrosine per GS subunit. P_I_ and P_II_ were the first and second peaks from a gel filtration column (Shapiro, 1969). P_I_ is a bifunctional enzyme (ATase) that adenylylates or deadenylylates GS (Jiang et al., 2007). P_II_ controls the activity of the ATase. We now know that P_II_ proteins are highly conserved and very widespread sensors used to transduce energy/carbon/nitrogen abundance signals in all domains of life (Kinch and Grishin, 2002; Sant'Anna et al., 2009). They are found in archaea, bacteria (Gram+ and Gram−), unicellular algae and plants. Many organisms have two or more genes for P_II_ proteins (reviewed in Forchhammer and Lüddecke, 2016), as *E. coli*, that has two paralogous genes encoding P_II_ proteins with distinct functions, one (GlnB) involved in the control of GS, and the other one (GlnK) being involved in the regulation of ammonia entry into the cell. By binding to target proteins, including channels, enzymes, or molecules involved in gene regulation and by altering the function of these target molecules, P_II_ proteins can regulate ammonia entry, nitrogen metabolism and gene expression (Forchhammer, 2008; Llácer et al., 2008). Cyanobacteria, and particularly among them *Synechococcus elongatus* PCC 7942 (hereafter *S. elongatus*), have been and continue to be very useful organisms for studies of P_II_ actions, fuelling structural understanding of P_II_ regulation. Studies on these organisms exemplify very clearly how enzyme activity and gene regulation can be controlled by P_II_ via formation of several complexes (summarized in Figure 1) mediated by weak intermolecular interactions that are crucially regulated by allosteric effectors of the proteins involved in these complexes. This is the focus of the present review.

**Figure 1:**
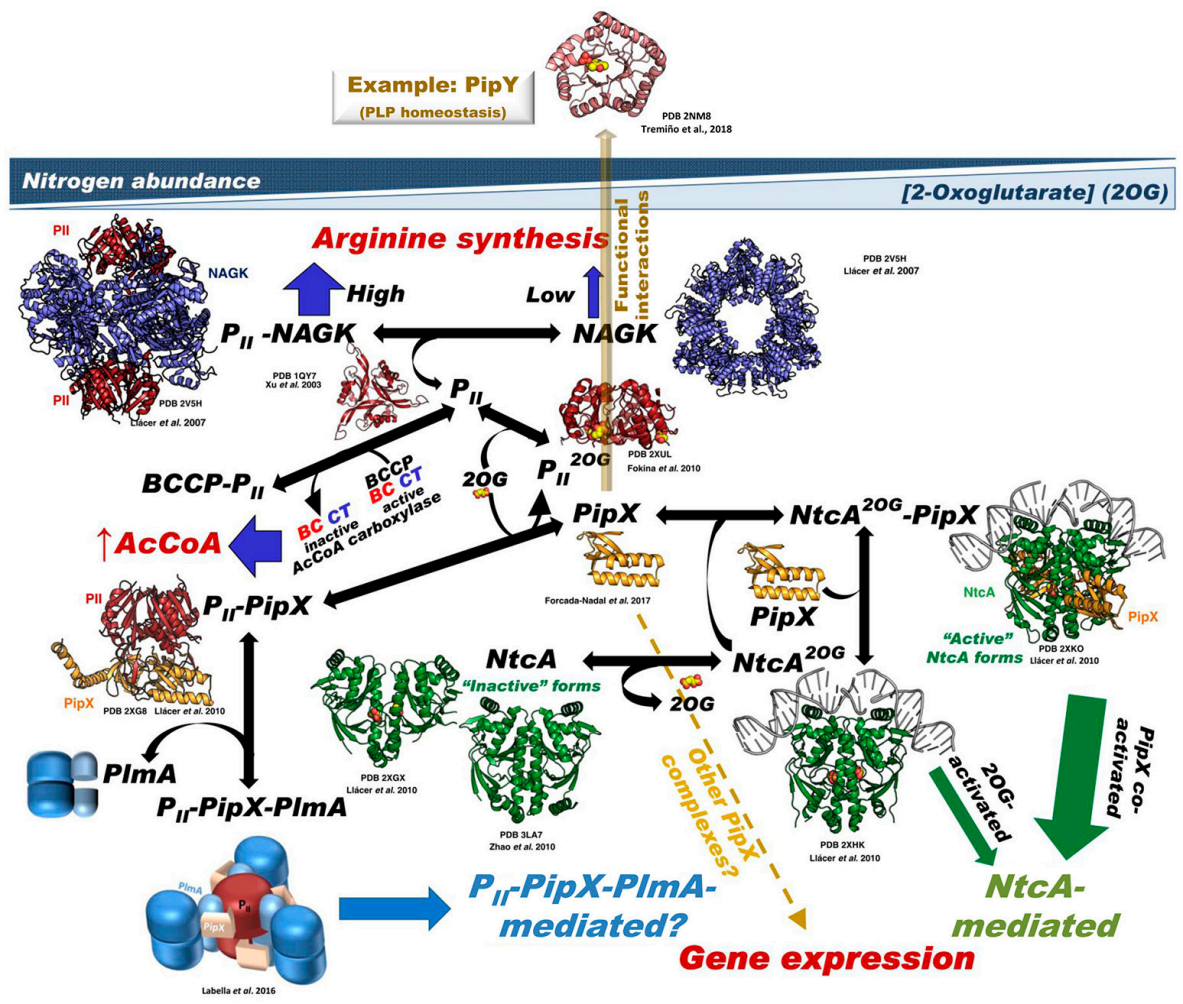
Summary of the P_II_-PipX-NtcA network of *S. elongatus*. The network illustrates its different elements and complexes depending on nitrogen abundance (inversely related to 2OG level) and the structures of the macromolecules and complexes formed (when known). For PlmA (dimer in darker and lighter blue hues for its dimerization and DNA-binding domains, respectively) and its complex the architectural coarse model proposed (Labella et al., 2016) is shown, with the C-terminal helices of PipX (schematized in the extended conformation) pink-colored and the two P_II_ molecules in dark red. The DNA complexed with NtcA and with NtcA-PipX is modeled from the structure of DNA-CRP (Llácer et al., 2010), since no DNA-NtcA structure has been reported. BCCP, biotin carboxyl carrier protein of bacterial acetyl CoA carboxylase (abbreviated AcCoA carboxylase); the other two components of this enzyme, biotin carboxylase and carboxyl transferase are abbreviated BC and CT, respectively. No structural model of BCC has been shown because the structure of this component has not been determined in *S. elongatus* and also because the structures of this protein from other bacteria lack a disordered 77-residue N-terminal portion that could be highly relevant for interaction with P_II_. The yellow broken arrow highlights the possibility of further PipX interactions not mediated by NtcA or P_II_-PlmA resulting in changes in gene expression (Espinosa et al., 2014). The solid semi-transparent yellowish arrow emerging perpendicularly from the flat network symbolizes the possibility of functional interactions of PipX not mediated by physical contacts between the macromolecules involved in the interaction, giving as an example the functional interaction with PipY. Its position outside the network tries to express the different type of interaction (relative to the physical contacts shown in the remainder of the network) as well as to place it outside the field of 2OG concentrations.

## The P_II_ signaling protein

**Figure 2:**
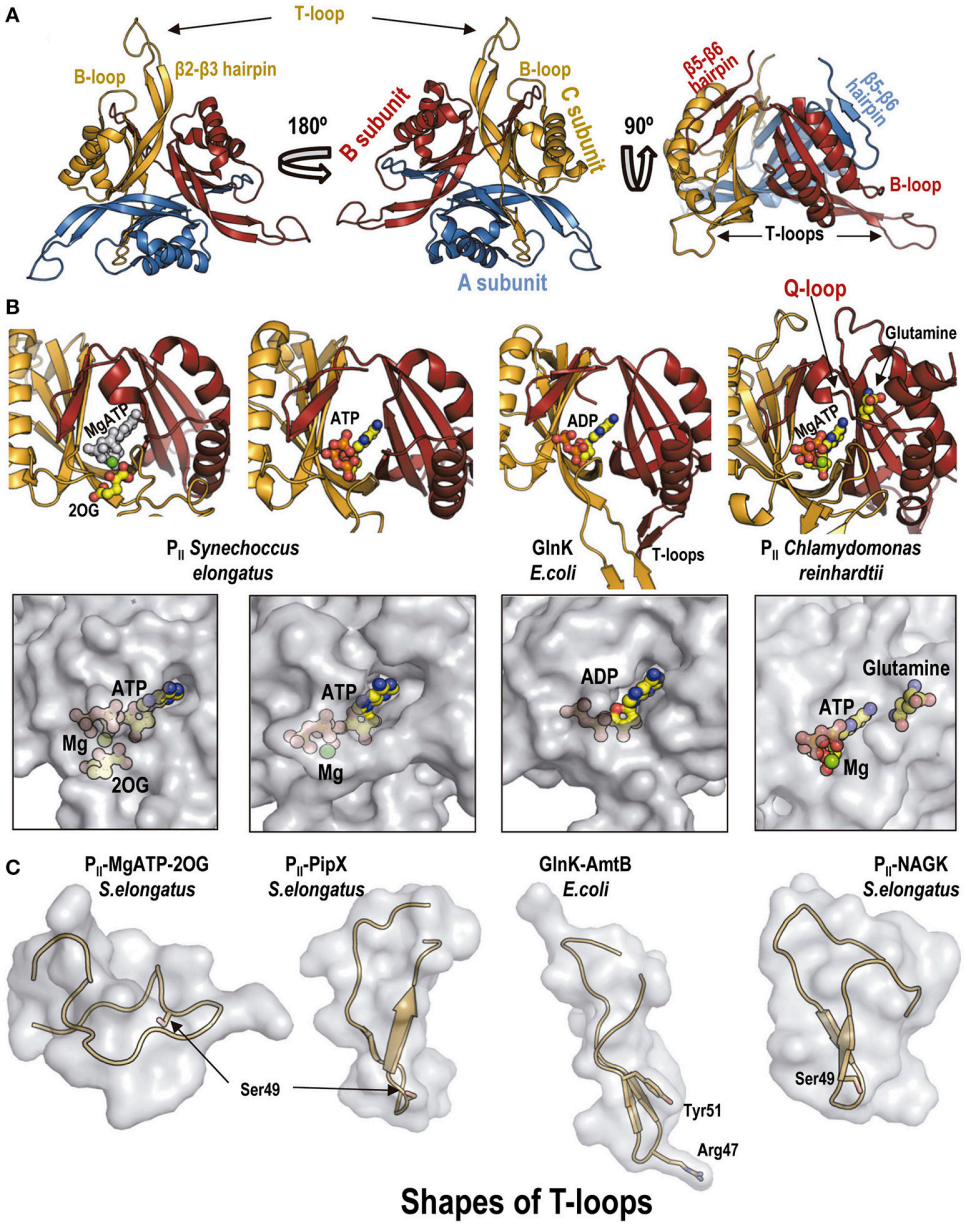
The P_II_ protein. **(A)** Overall view in cartoon representation of *S. elongatus* P_II_ along its three-fold axis from the P_II_ flat face (left), from its convex side (middle), or with the three-fold axis vertical and the flat surface down (right). The structure corresponds to Protein Database (PDB) file 1QY7 (Xu et al., 2003). Each subunit in the trimer is colored differently. Some relevant traits are highlighted. **(B)** The P_II_ allosteric sites shown in cartoon representation (top) and in semi-transparent zoomed surface representation (bottom) in approximately the same pose as in the cartoon representation. For clarity, in the cartoon representations only the two subunits forming each site are shown, in the same colors as in **(A)**. Ligands are shown in sticks and balls representation, with atoms of C, O, N, P, and Mg in yellow, red, blue, orange, and green, respectively, except in the leftmost cartoon figure in which all the atoms of ATP are pale gray to highlight the bound 2OG (colored). Note in the corresponding panel of the bottom row that MgATP and 2OG are nearly fully buried in the P_II_ molecule. The organisms from which the P_II_ derive are indicated in the figure. The two panels to the left belong to isolated *S. elongatus* P_II_ (PDB file 2XZW; Fokina et al., 2010a); the third panels illustrate *E. coli* GlnK taken from its complex with AmtB (PDB 2NUU, Conroy et al., 2007); the rightmost panels show *Chlamydomonas reinhardtii* P_II_, taken from its complex with *Arabidopsis thaliana* NAGK (PDB 4USJ; Chellamuthu et al., 2014). **(C)** Illustration of different shapes of the T-loops found in distinct complexes with allosteric effectors or with partner proteins. The T-loop is shown in cartoon representation, within a semi-transparent surface representation as if this loop were isolated from the remainder of P_II_ and from the protein partner in the complex. In the third panel, the side chain of Arg47 of *E. coli* GlnK is represented in sticks, given its importance for inhibiting the AmtB channel. Taken, from left to right, from: *S. elongatus* P_II_ with MgATP and 2OG bound (PDB file 2XZW; Fokina et al., 2010a); *S. elongatus* P_II_-PipX (PDB 2XG8; Llácer et al., 2010); *E. coli* GlnK-AmtB complex (PDB 2NUU, Conroy et al., 2007); and P_II_-NAGK complex (PDB 2V5H; Llácer et al., 2007).

*S. elongatus* P_II_ (Figure 2), as other P_II_ proteins, is a homotrimer of a polypeptide chain of 112 amino acids that exhibits the ferredoxin fold (β*αβ*)_2_ followed by a beta hairpin (Xu et al., 2003). The trimer (Figure 2A) has a hemispheric body nucleated by three antiparallel oblique (relative to the three-fold axis) β-sheets, each one formed by the 4-stranded sheet (topology ↓β2↑β3↓β1↑β4) of a subunit (see for example subunit B in the central panel of Figure 2A) extended on its β4 end by the C-terminal hairpin (β5-β6) of an adjacent subunit (subunit A) and on the β2 end by the β2-β3 hairpin stem (the root of the T-loop, see below) of the other subunit of the trimer (subunit C). The three sheets become continuous on the flat face of the hemispheric body via their β2-β3 hairpins (Figure 2A). The subunit sheets encircle like a 3-sided pyramid the three-fold axis, filling the inner space between them with their side-chains. They are covered externally by 6 helices (two per subunit) that run parallel to the β strands, contributing to the rounded shape of the hemispheric trimer (Figure 2A, panel to the right) and to the outer part of its equatorial flat face. In the convex face, three crevices are formed at subunits junctions between adjacent β-sheets, over the β2-β3 hairpins (Figure 2B). These crevices host the sites for the allosteric effectors ATP/ADP [and in some species AMP (Palanca et al., 2014)] and 2-oxoglutarate (2OG) that endow P_II_ with its sensing roles (Kamberov et al., 1995; Zeth et al., 2014) (Figure 2B), the nucleotides reflecting the energy status (Fokina et al., 2011) and 2OG reflecting the abundance of carbon and, inversely, the nitrogen richness (see for example Muro-Pastor et al., 2001).

Very salient structural features of P_II_ are the long flexible T-loops (Figures 2A,C) formed by the 18 residues that tip the β2-β3 hairpin of each subunit (Xu et al., 2003). These loops are key elements (although not the exclusive ones, see Rajendran et al., 2011 and Schumacher et al., 2015) for P_II_ interaction with its targets (Conroy et al., 2007; Gruswitz et al., 2007; Llácer et al., 2007, 2010; Mizuno et al., 2007; Zhao et al., 2010b; Chellamuthu et al., 2014). By binding at the boundary between the T-loop and the P_II_ body, at the crevice formed between adjacent subunits, the adenine nucleotides and MgATP/2OG promote the adoption by the T-loop of different conformations (Figure 2C) (Fokina et al., 2010a; Truan et al., 2010; Maier et al., 2011; Zeth et al., 2014) that favor or disfavor P_II_ binding to a given P_II_ target.

The T-loop also is the target of regulatory post-translational modification (reviewed in Merrick, 2015), first recognized in the regulatory cascade of the GS of *E. coli* as uridylylation of Tyr51 (see Figure 2C, 3rd panel from the left) mediated by a glutamine-regulated bifunctional P_II_ uridylylating-deuridylylating enzyme, GlnD (Stadtman, 2001). Thus, in the enterobacterial GS regulating cascade P_II_ is uridylylated or deuridylylated depending on whether 2-OG is abundant and L-glutamine is low or the reverse. P_II_-UMP activates the GS deadenylylating activity of ATase (Jiang et al., 2007), activating GS by decreasing its susceptibility to feed-back inhibition (Stadtman, 2001). This uridylylation (or in Actinobacteria adenylylation of Tyr51) occurs at least in proteobacteria and actinobacteria (Merrick, 2015), but it might be more widespread, since it has also been reported in an archaeon (Pedro-Roig et al., 2013). Structural studies with *E. coli* P_II_ (Palanca and Rubio, 2017) have excluded the stabilization of the T-loop into a fixed conformation by Tyr51 uridylylation, suggesting that the Tyr51-bound UMP physically interacts with the ATase. Although Tyr51 is conserved in cyanobacteria, it is not uridylylated. The T-loop serine 49 (Figure 2C, 1st, 2nd and 4th panels from the left) is phosphorylated in *S. elongatus* under conditions of nitrogen starvation by an unknown mechanism (Forchhammer and Tandeau de Marsac, 1994), whereas the phosphatase that dephosphorylates phosphoSer49 has been identified and proven to be 2OG-sensitive (Irmler et al., 1997).

FRET studies with engineered fluorescent *S.elongatus* P_II_ used as an ADP and ATP-sensitive probe (Lüddecke and Forchhammer, 2015) have challenged the claim (Radchenko et al., 2013) that P_II_ proteins have a very slow ATPase activity that would regulate P_II_ similarly as the signaling GTPases with bound GTP and GDP. Although this ATPase was reported as a 2OG-triggered switch that appeared an intrinsic trait of P_II_ proteins (Radchenko et al., 2013), the FRET experiments with *S. elongatus* P_II_ (Lüddecke and Forchhammer, 2015) appear to indicate that an endogenous ATPase is not a relevant mechanism for the transition of P_II_ into the ADP state.

## P_II_ complexes with channels

**Figure 3:**
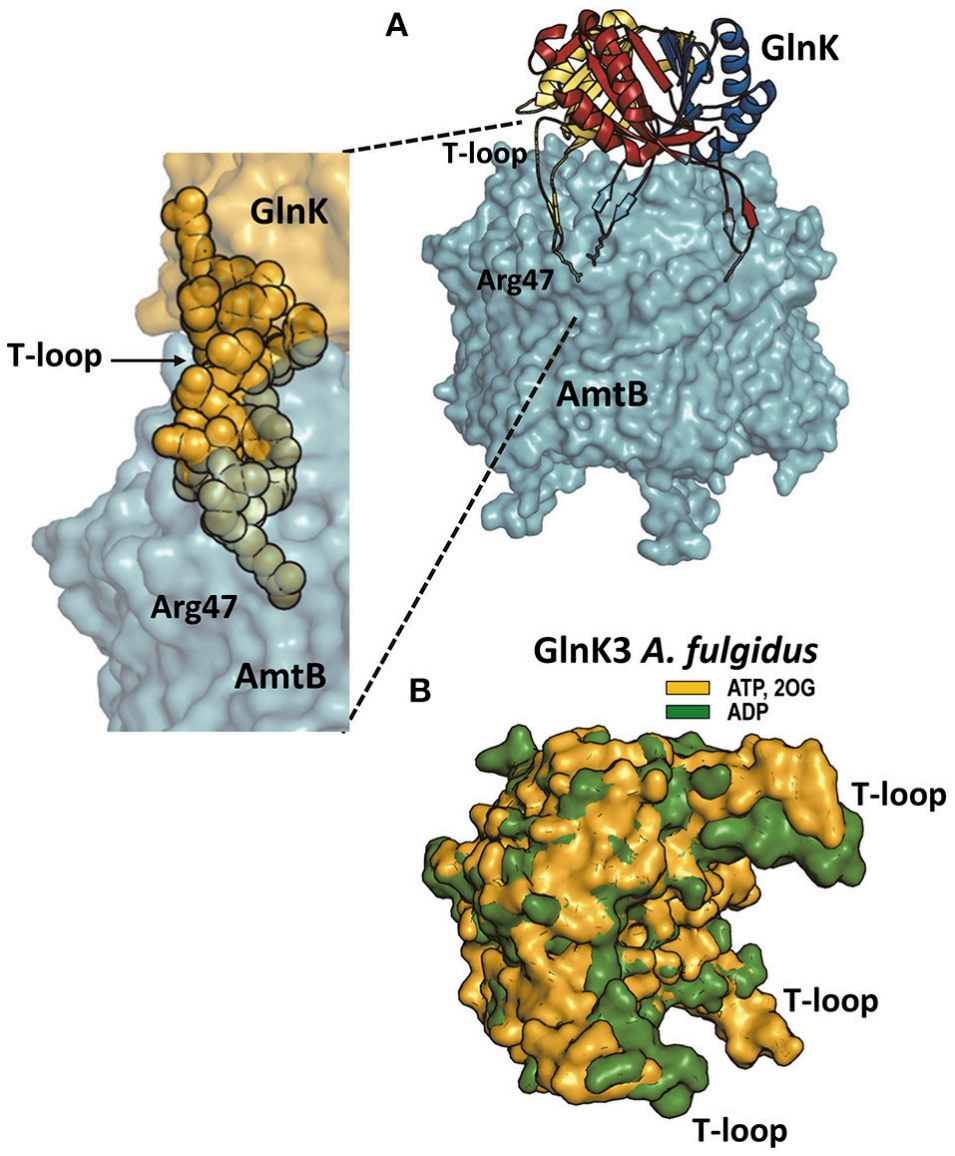
P_II_ proteins and the ammonia channel. **(A)** The structure (PDB file 2NUU; Conroy et al., 2007) of the *E. coli* complex of GlnK (a P_II_ protein in charge of ammonia channel regulation) and the ammonia channel AmtB is shown to the right, whereas the zoom to the lower left shows only a part of the complex, to highlight the interaction of one T-loop with one channel. AmtB is in semi-transparent surface representation. GlnK is in the main figure in cartoon representation with each subunit colored differently, with the side-chain of the T-loop residue Arg47 shown in sticks representation. In the zoomed image GlnK is shown in surface representation in yellow with the T-loop residues highlighted in space-filling representation, illustrating the fact that the side-chain of Arg47 is the element getting deep into the channel and blocking it. **(B)** Super-imposition of the structures of *Archeoglobus fulgidus* GlnK3 (one of the three P_II_ proteins of the GlnK type in this archaeon; Maier et al., 2011) with ADP bound (green; PDB file code 3TA1) or with ATP and 2OG bound (yellow; PDB 3TA2) to illustrate how 2OG binding fixes the T-loops in an outwards-flexed position (relative to the positions without 2OG) that would be inappropriate for fitting the topography of the entry chambers to the three ammonia channels in trimeric Amt (the ammonia channel in this organism).

The first structurally solved P_II_ complex was the one of *E. coli* GlnK with the AmtB ammonia channel (Conroy et al., 2007; Gruswitz et al., 2007) (Figure 3A) formed under nitrogen richness conditions. This structure showed that AmtB was inhibited by GlnK because the extended T-loop fits the channel entry, with the insertion into the channel of a totally extended arginine emerging from the T-loop and blocking the channel space (Figure 2C, 3rd panel from the left, and Figure 3A, zoom). The ADP-bound and MgATP/2OG-bound structures of an *Archeoglobus fulgidus* GlnK protein (Maier et al., 2011) indicated that 2OG may prevent GlnK binding to the ammonia channel because of induced flexing outwards (relative to the 3-fold molecular axis) of the T-loops, preventing their topographical correspondence with the three holes of the trimer of ammonia channels (Figure 3B). Interestingly, the T-loops of MgATP/2OG-bound *S. elongatus* P_II_ (Figure 2C, leftmost panel) and *A. fulgidus* GlnK3 (Figure 3B) exhibited different flexed conformations (relative to the ADP-bound extended forms), and thus 2OG-binding by itself does not determine a single T-loop conformation, at least with different P_II_ proteins.

Yeast two hybrid approaches (Osanai et al., 2005) detected the interaction between P_II_ and the putative channel PamA (encoded by *sll0985*) of *Synechocystis* sp. PCC 6803 (from now on *Synechocystis*), but molecular detail on this protein is non-existent, and, therefore, it is uncertain whether such interaction might resemble the GlnK-AmtB interaction. PamA is not conserved in many cyanobacteria, and the most closely related putative protein of *S. elongatus*, the product of the *Synpcc7942_0610* gene, failed to give interaction signal with *S. elongatus* P_II_ in yeast two hybrid assays (Castells, M.A., PhD Dissertation, Universidad de Alicante, 2010), despite the fact that the sequence identity with PamA concentrated in the C-terminal region, where P_II_ binds in *Synechocystis* (Osanai et al., 2005). *In vitro* studies with the recombinantly produced *Synechococystis* PamA and P_II_ showed that their interaction was lost in the presence of ATP and 2OG. Thus, similarly to the GlnK-AmtB and GlnK3-Amt complexes, the P_II_-PamA complex is formed under conditions of nitrogen abundance. However, T-loop phosphorylation did not dissociate this complex (Osanai et al., 2005). The function of PamA is not known, but its deletion from *Synechocystis* changed the expression of some NtcA-dependent genes (Osanai et al., 2005) by unclarified mechanisms.

## P_II_ complexes with enzymes in cyanobacteria (and beyond)

**Figure 4:**
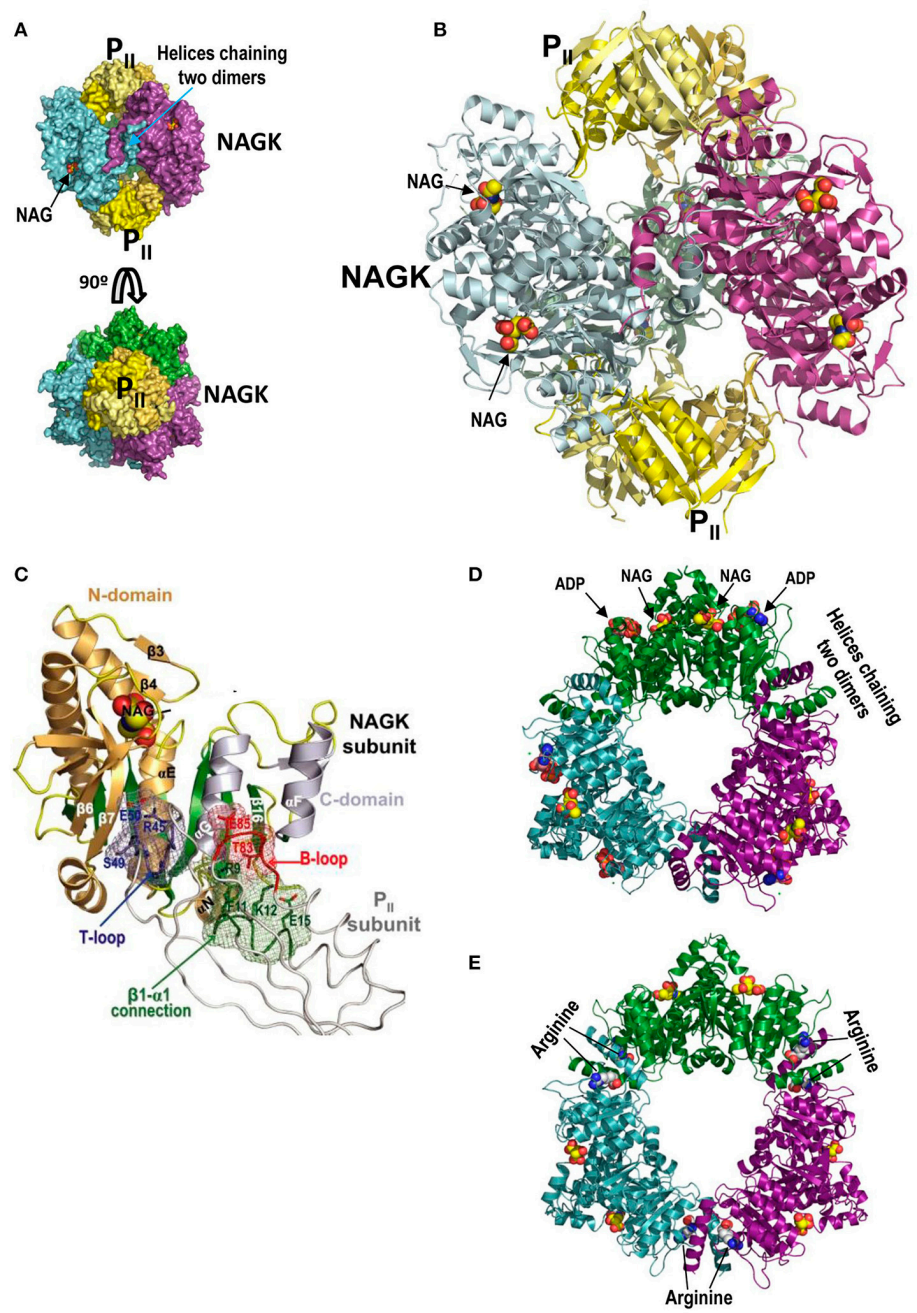
P_II_-NAGK complex and active and arginine-inhibited NAGK. **(A)** The P_II_-NAGK complex of *S. elongatus* (PDB 2V5H; Llácer et al., 2010). Surface representations of the complex formed by two P_II_ trimers (yellow) capping on both ends the doughnut-like NAGK hexamer (trimer of dimers; each dimer in a different color). The three-fold axis is vertical (top) or perpendicular to the page (bottom). Figure of J.L. Llácer and V. Rubio taken from Chin (2008). Reprinted with permission from AAAS **(B)**. Cartoon representation of the *S. elongatus* P_II_-NAGK complex after removing the back NAGK dimer for clarity. The three-fold symmetry axis is vertical. Reprinted from Current Opinion in Structural Biology, 18, Llácer et al., Arginine and nitrogen storage, 673–681, 2008, with permission from Elsevier. **(C)** P_II_ subunit-NAGK subunit contacts. P_II_, NAGK, and NAG are shown as strings, ribbons, and spheres, respectively. The contacting parts of the T-loop, B-loop, and β1–α1 connection, including some interacting side chains (in sticks), are blue, red, and green, respectively. The surfaces provided by these elements form meshworks of the same colors. The NAGK central β-sheet is green, and other β-strands and the α-helices are brownish and grayish for N- and C-domains, respectively. Some NAGK elements and P_II_ residues are labeled. This figure and its legend reproduce with some modifications a figure and its legend of Llácer et al. (2007). The crystal structure of the complex of P_II_ and acetylglutamate kinase reveals how P_II_ controls the storage of nitrogen as arginine. Copyright (2007) National Academy of Sciences. **(D,E)**, active and inactive conformations, respectively, of hexameric arginine-inhibitable NAGK. The active form is from a crystal of the enzyme from *Pseudomonas aeruginosa* (PDB 2BUF) while the inactive form is from the *Thermotoga maritima* enzyme (PDB 2BTY) (Ramón-Maiques et al., 2006). Note that the inactive form is widened relative to the active form, and that it has arginine sitting on both sides of each interdimeric junction. In the active form the nucleotide (in this case the product ADP rather than the substrate ATP) and NAG sit one in each domain of individual subunits. The NAGK observed in the P_II_-NAGK complex is in the active form, being stabilized in this form by its contacts with P_II_.

The complexes of P_II_ with the N-acetyl-L-glutamate kinase (NAGK) enzymes from *S. elongatus* and *Arabidopsis thaliana* presented a very different architecture with respect to the structure of the GlnK-AmtB complex of *E. coli* (Llácer et al., 2007; Mizuno et al., 2007) (Figures 4A,B). The P_II_-NAGK complex is an activating complex in which the T-loops of P_II_ are flexed (Figure 2C, rightmost panel) and integrated into a hybrid (both proteins involved) β-sheet with NAGK, forming also a hybrid ion-pair network (Figure 4C; Llácer et al., 2007). Apparently this flexing from an extended conformation could occur in two steps (Fokina et al., 2010b). The initial step would be mediated by a smaller loop of P_II_ called the B-loop (Figures 2A, 4C). P_II_ binding of 2OG also favors the flexing of the T-loop (Figure 2C, leftmost panel) (Fokina et al., 2010a; Truan et al., 2010) although the resulting conformation appears inappropriate for interacting with NAGK. In addition, 2OG can also promote the disassembly of the P_II_-NAGK complex because certain P_II_ residues like Arg9 that are involved in the binding of 2OG are also involved in the interaction with NAGK (and also with PipX, another target of P_II_, see below). Therefore, 2OG, an indicator of low ammonia levels (Muro-Pastor et al., 2001), abolishes P_II_-NAGK complex formation (Maheswaran et al., 2004) (Figure 1). In *S. elongatus* 2OG can also promote the disassembly of the P_II_-NAGK complex by favoring the phosphorylation of Ser49 (Forchhammer and Tandeau de Marsac, 1994; Irmler et al., 1997), since the bound phosphate sterically prevents formation of the P_II_-NAGK hybrid β-sheet (Llácer et al., 2007).

Plants and cyanobacteria stockpile ammonia as arginine, the protein amino acid with the largest nitrogen content (four atoms per arginine molecule). Arginine-rich proteins are very abundant in plant seeds (VanEtten et al., 1963). Cyanobacteria make non-ribosomally an arginine-rich amino acid polymer called cyanophycin (Oppermann-Sanio and Steinbüchel, 2002; Watzer and Forchhammer, 2018). The arginine stockpiling as arginine-rich macromolecules minimizes the osmotic effect while permitting rapid nitrogen mobilization for protein-building processes such as seed germination and cell multiplication. The selection of NAGK as the regulatory target stems from the fact that in many bacteria (including cyanobacteria) and in plants NAGK controls arginine synthesis via feed-back inhibition by L-arginine (Hoare and Hoare, 1966; Cunin et al., 1986; Lohmeier-Vogel et al., 2005; Beez et al., 2009). This inhibition must be overcome if large amounts of ammonia have to be stored as arginine (Llácer et al., 2008). Indeed, the P_II_-NAGK complex exhibits decreased inhibition by arginine (Maheswaran et al., 2004; Llácer et al., 2008).

In arginine-sensitive NAGK (Figures 4D,E) the N-terminal α-helix of each subunit interacts with the same helix of an adjacent dimer, chaining three NAGK homodimers into a doughnut-shaped hexameric ring with three-fold symmetry and a central large hole (Ramón-Maiques et al., 2006). The NAGK reaction (phosphorylation of the γ-COOH of N-acetyl-L-glutamate, NAG, by ATP) occurs within each NAGK subunit. NAG and ATP sit over the C-edge of the central 8-stranded largely parallel β sheet of the N-terminal and C-terminal domains, respectively (Figure 4D; Ramón-Maiques et al., 2002). Catalysis requires the mutual approach of both domains of each subunit to allow the contact of the ATP terminal phosphate with the attacking NAG γ-COOH (Ramón-Maiques et al., 2002; Gil-Ortiz et al., 2003). Arginine, by binding in each subunit next to the N-terminal α-helix (Figure 4E), expands the hexameric ring hampering the contact of the reacting groups and preventing catalysis (Ramón-Maiques et al., 2006).

In the P_II_-NAGK complex two P_II_ trimers sit on the three-fold axis of the complex, one on each side of the NAGK ring, making contacts with the inner circumference of this ring (Figures 4A,B). Each P_II_ subunit interacts via its T and B loops with each NAGK subunit (Figure 4C) gluing the two domains of this last subunit (Figure 4A). By restricting NAGK ring expansion (Figure 4C) even when arginine is bound, P_II_ renders NAGK highly active (Llácer et al., 2007; Mizuno et al., 2007). P_II_ does not compete physically with arginine for its sites on NAGK, simply these sites are widened in the P_II_-NAGK complex (Llácer et al., 2007), resulting in decreased apparent affinity of NAGK for arginine (as reflected in the dependency of the NAGK activity on the arginine concentration). In addition, the hybrid P_II_-NAGK ion pair network (Figure 4C) enhances the apparent affinity for NAG (assessed as the K_m_ or S_0.5_ value of NAGK for NAG) of cyanobacterial NAGK (Maheswaran et al., 2004; Llácer et al., 2007). Overall, the NAGK bound to P_II_ exhibits decreased apparent affinity for arginine and increased activity, rendering NAGK much more active in the presence of arginine than when not bound to P_II_ (Llácer et al., 2008), something that is crucial for nitrogen storage as arginine.

NAGK appears to be a P_II_ target only in organisms performing oxygenic photosynthesis (cyanobacteria, algae, and plants, Burillo et al., 2004). P_II_ proteins from plants have lost the ability to bind ADP, while still binding ATP and 2OG (Lapina et al., 2018). In addition, except in *Brassicae*, the C-terminal part of plant P_II_ is extended to form two helical segments and a connecting loop (Q-loop; Figure 2B, rightmost panels), creating a novel glutamine site, resulting in glutamine-sensitivity of the P_II_-NAGK interaction (Chellamuthu et al., 2014). This is not the case with cyanobacterial P_II_, which binds both ADP and ATP and is glutamine-insensitive (Chellamuthu et al., 2014).

P_II_ has also been shown to interact in plants (Feria-Bourrellier et al., 2010) and bacteria, including cyanobacteria (Rodrigues et al., 2014; Gerhardt et al., 2015; Hauf et al., 2016), with the biotin carboxyl carrier protein (BCCP) of the enzyme acetyl coenzyme A carboxylase (AcCoA carboxylase) (Figure 1), although this complex has not been characterized structurally. BCCP is the component that hosts the covalently bound biotin that shuttles between the biotin carboxylase component and the transferase component of AcCoA carboxylase (Rubio, 1986). P_II_-BCCP complex formation tunes down AcCoA utilization and thus subsequent fatty acid metabolism (Feria-Bourrellier et al., 2010; Gerhardt et al., 2015; Hauf et al., 2016), promoting uses of AcCoA for different purposes than the synthesis of fatty acids, and therefore linking P_II_ to AcCoA and fatty acid metabolism. For interaction, P_II_ has to be in the ATP-bound and 2OG-free form (Figure 1) (Gerhardt et al., 2015; Hauf et al., 2016), which are conditions at which P_II_ also binds to NAGK (Llácer et al., 2008). Therefore, there could be *in vivo* simultaneous activation of NAGK and inhibition of AcCoA carboxylase by P_II_. Mutational evidence suggests the involvement of the T-loop in this interaction with AcCoA carboxylase (Hauf et al., 2016), in principle excluding P_II_-NAGK-BCCP ternary complex formation and raising the possibility of competition between NAGK and BCCP for P_II_.

The classical example of interaction of P_II_ with an enzymatic target was with the ATase of *E. coli* (see introductory section), with which uridylylated or deuridylylated GlnB (GlnB is one of the two P_II_ proteins of *E. coli*) can interact (Stadtman, 2001). We will not deal with this enzyme here because the P_II_/ATase/GS cascade of enterobacteria does not appear to have general occurrence, for example in cyanobacteria, and also because we have only partial information on the structure of the ATase (Xu et al., 2004, 2010) and no direct information on the structure of the GlnB-ATase complex, although a model for such complex has been proposed (Palanca and Rubio, 2017).

## The PipX adaptor protein and its complex with P_II_

A yeast two hybrid search for proteins interacting with P_II_ in *S. elongatus* identified (Burillo et al., 2004), in addition to NAGK, a small novel protein (89 amino acids) that was named PipX (P_II_-interacting protein X). This protein was identified later in a search (Espinosa et al., 2006) for proteins interacting with NtcA, the global nitrogen regulator of cyanobacteria (Vega-Palas et al., 1992). PipX binding to P_II_ occurs under conditions of ammonia abundance (Figure 1), the same conditions prevailing for P_II_-NAGK complex formation (Espinosa et al., 2006). NAGK-PipX competition for P_II_ was revealed in NAGK assays that showed that PipX decreased P_II_-activation and increased arginine inhibition of NAGK (Llácer et al., 2010), excluding NAGK-P_II_-PipX ternary complex formation. 2OG binding to P_II_ disassembles the P_II_-PipX complex (Espinosa et al., 2006; Llácer et al., 2010), leaving PipX free to interact with NtcA (Figure 1).

**Figure 5:**
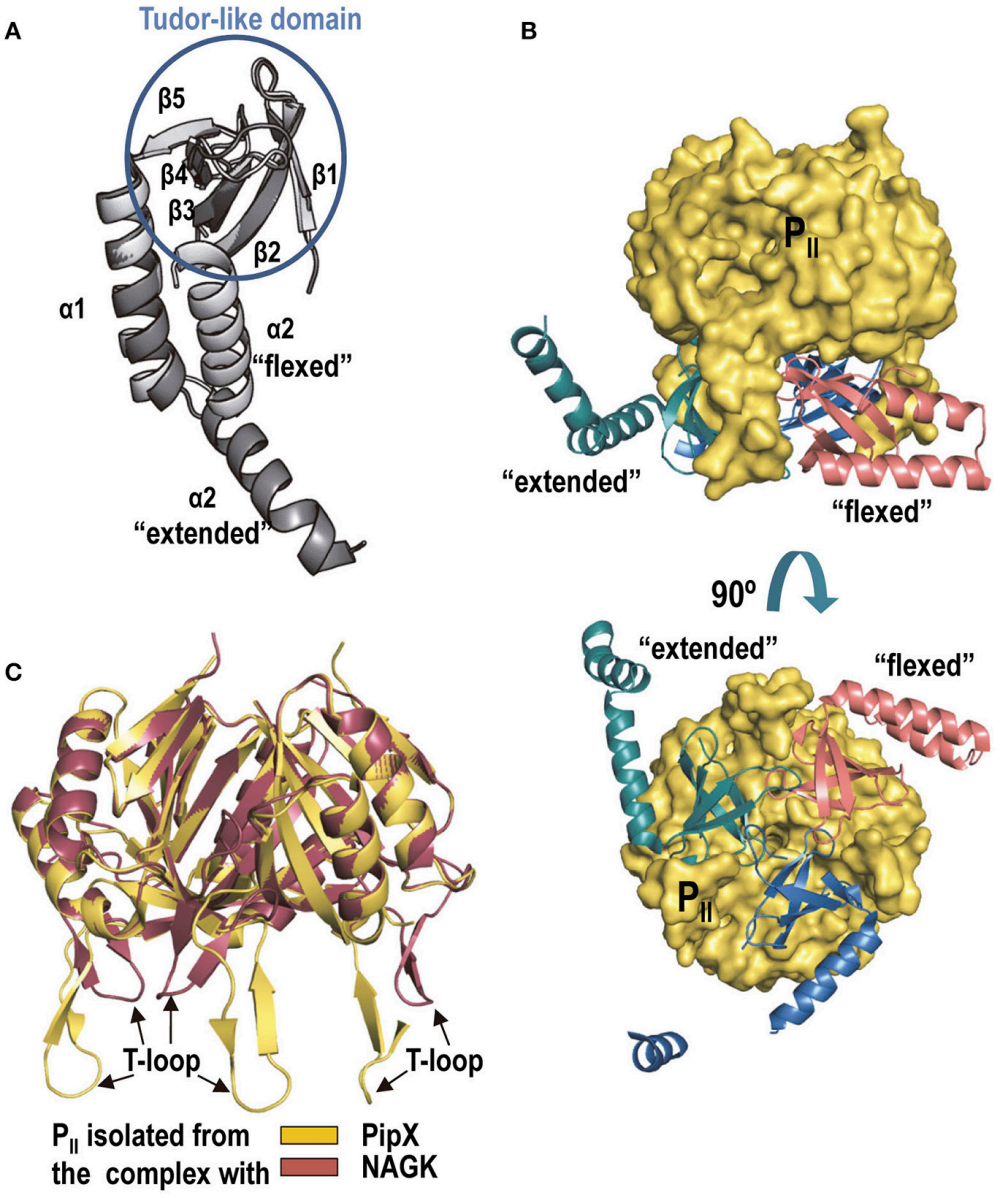
PipX and the P_II_-PipX complex. **(A)** The structures of PipX in the flexed conformation and in one of the two extended conformations observed in the *S. elongatus* P_II_-PipX complex are shown (extracted from PDB 2XG8; Llácer et al., 2010). Note that the major difference between the two conformations is the large movement of the C-terminal helix around its flexible linker (Forcada-Nadal et al., 2017). The same flexed conformation was observed in the complex with NtcA (see below) and agrees with the data of structural NMR studies on isolated PipX (Forcada-Nadal et al., 2017). The elements of the Tudor-like domain are encircled in a blue circumference. **(B)** The P_II_-PipX complex of *S. elongatus* viewed with the three-fold axis of PII vertical (top) or in a view along this axis, looking at the flat face of P_II_ (bottom). **(C)** Superimposition of *S. elongatus* P_II_ in the complex with PipX and in that with NAGK. The changes in the T-loops are very patent.

The crystal structures of P_II_-PipX complexes of *S. elongatus* (Llácer et al., 2010) and of *Anabaena* sp. PCC7120 (Zhao et al., 2010b) provided the first structural information on PipX (Figure 5A), revealing that it is formed by a compact body folded as a Tudor-like domain (a horseshoe-curved β-sheet sandwich) (Lu and Wang, 2013), followed by two C-terminal helices. In the P_II_-PipX complex (Figure 5B) the three PipX molecules are enclosed in a cage formed between the flat face of the hemispheric P_II_ trimer and its three fully extended T-loops (see Figure 2C, 2nd panel from the left) emerging perpendicularly to the P_II_ flat face at its edge. The shape and orientation of these T-loops is very different relative to the P_II_ bound to NAGK, Figure 5C). In turn, the caged Tudor-like domains form a homotrimer over the P_II_ flat surface (Figure 5B, bottom), with the PipX self-interaction detected in yeast three-hybrid assays using P_II_ as bridging protein (Llácer et al., 2010). Tudor-like domains characteristically interact with RNA polymerase (Steiner et al., 2002; Deaconescu et al., 2006; Shaw et al., 2008), suggesting that PipX could have some role in gene expression that would be blunted by sequestration of these domains in the P_II_ cage.

In the structure of the *Anabaena* P_II_-PipX complex, the two C-terminal helices of each PipX molecule lie one along the other in antiparallel orientation (“flexed”), being exposed between two adjacent T-loops in transversal orientation relative to these loops (Zhao et al., 2010b). Recent structural NMR data on isolated PipX showed that when PipX is alone (that is, not bound to a partner) the C-terminal helices are “flexed” (Figure 5A) (Forcada-Nadal et al., 2017). As shown below, the C-terminal helices of PipX in the NtcA-PipX complex are also flexed (Llácer et al., 2010). However, in the *S. elongatus* P_II_-PipX complex only one PipX molecule presents the “flexed” conformation, whereas in the other two PipX molecules the C-terminal helix is “extended,” not contacting the previous helix and emerging centrifugally outwards from the complex, between two T-loops (Figure 5B) (Llácer et al., 2010). P_II_ binding might facilitate the extension of the PipX C-terminal helix, endowing the P_II_-PipX complex with a novel surface and novel potentialities for interaction with other components. These novel potentialities were substantiated recently by the identification, in yeast three-hybrid searches (Labella et al., 2016), of interactions of PipX in the P_II_-PipX complex with the homodimeric transcription factor PlmA (see proposal for the architecture of this complex in Figure 1 bottom left; Labella et al., 2016). Interactions were not observed in yeast two-hybrid assays between PipX or P_II_ and PlmA. Residues involved in three-hybrid interactions, mapped by site-directed mutagenesis, are largely localized in the C-terminal helix of PipX. PlmA belongs to the GntR super-family of transcriptional regulators, but is unique to cyanobacteria (Lee et al., 2003; Hoskisson and Rigali, 2009; Labella et al., 2016). Little is known about PlmA functions other that it is involved in plasmid maintenance in *Anabaena* sp. strain PCC7120 (Lee et al., 2003), in photosystem stoichiometry in *Synechocystis* sp. PCC6803 (Fujimori et al., 2005), in regulation of the highly conserved cyanobacterial sRNA YFR2 in marine picocyanobacteria (Lambrecht et al., 2018), and that it is reduced by thioredoxin, without altering its dimeric nature in *Synechocystis* sp. PCC6803 (Kujirai et al., 2018). The P_II_-PipX-PlmA ternary complex suggests that PipX can influence gene expression regulation via PlmA, although the PlmA regulon remains to be defined.

## The gene expression regulator NtcA

**Figure 6:**
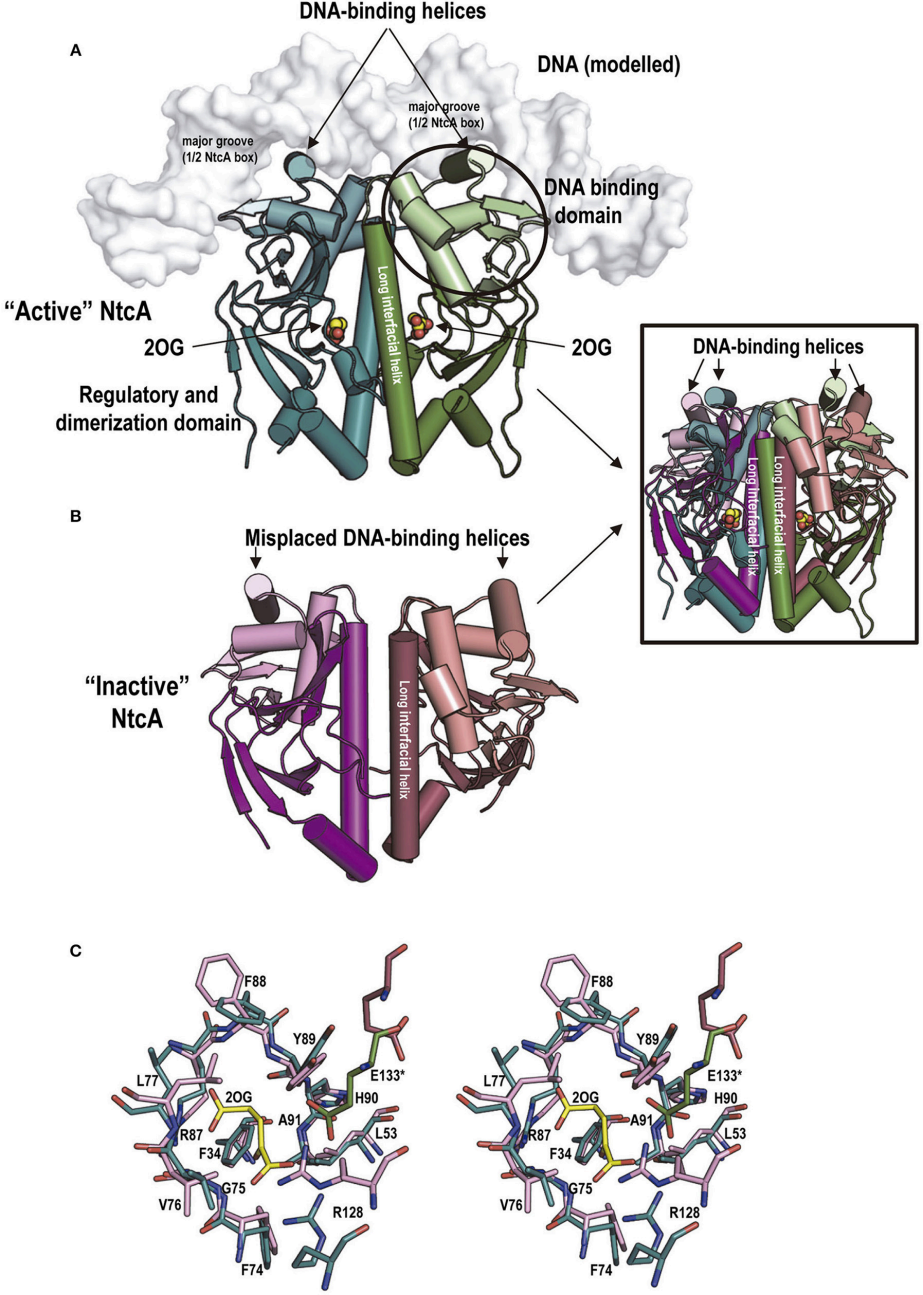
NtcA structure, 2OG binding to it and associated conformational changes. **(A,B)**, structures of “active” **(A)** and “inactive” **(B)** *S. elongatus* NtcA (PDB files 2XHK and 2XGX, respectively) (Llácer et al., 2010). The two subunits of each dimer are in different colors, with the DNA-binding domains in a lighter hue than the regulatory domain of the same subunit. In the cartoon representation used, helices are shown as cylinders to illustrate best the changes in position of the DNA binding helices and of the long interfacial helices (labeled) upon activation. Bound 2OG is shown in “active” NtcA (in spheres representation, with C and O atoms colored yellow and red, respectively). The DNA, in surface representation in white, has been modeled from the CRP-DNA structure (for details see Llácer et al., 2010). The inset superimposes the “active” and “inactive” forms colored as in the main figure to illustrate the magnitude of the changes. **(C)** Stereo view of sticks representation of the 2OG site residues of the “active” (green) and “inactive” (raspberry) forms of NtcA. The 2OG bound to the “active” form is distinguished by its yellow C atoms. Note that only two residues, both 2OG-interacting and highly polar, experience large changes in their positions between the inactive and the active forms: Arg128 from the long interfacial helix of the subunit that provides the bulk of the residues of the site, and Glu133 from the interfacial helix of the other subunit. They are believed to trigger the changes in the relations between the two interfacial helices that result in NtcA “activation”.

When ammonia becomes scarce the increasing 2OG levels should determine the disassembly of the P_II_-NAGK, P_II_-BCCP, and P_II_-PipX complexes (Figure 1). These same conditions promote the binding of PipX (see below) to the transcriptional regulator NtcA (Figure 1), an exclusive cyanobacterial factor of universal presence in this phylogenetic group (Vega-Palas et al., 1992; Herrero et al., 2001; Körner et al., 2003). The determination of the structures of NtcA from *S. elongatus* (Figures 6A,B) (Llácer et al., 2010) and from *Anabaena* sp. PCC7120 (Zhao et al., 2010a) confirmed the sequence-based inference (Vega-Palas et al., 1992) that NtcA is a homodimeric transcriptional regulators of the family of CRP (the cAMP-regulated transcriptional regulator of *E. coli*) (McKay and Steitz, 1981; Weber and Steitz, 1987). Similarly to CRP, NtcA has a C-terminal DNA binding domain of the helix-turn-helix type. In CRP, the DNA binding helices of its two C-terminal domains are inserted in two adjacent turns of the major groove of DNA that host the imperfectly palindromic target DNA sequence (called here the CRP box) (McKay and Steitz, 1981; Weber and Steitz, 1987). The consensus DNA sequence to which NtcA binds (consensus NtcA box) is quite similar to the consensus CRP box (Berg and von Hippel, 1988; Luque et al., 1994; Jiang et al., 2000; Herrero et al., 2001; Omagari et al., 2008), and thus NtcA and CRP are expected to bind in similar ways to their target DNA sequences.

*In vitro* studies revealed that 2OG is an NtcA activator (Tanigawa et al., 2002; Vázquez-Bermúdez et al., 2002), increasing NtcA affinity for its target sequences. As in the case of cAMP for CRP, 2OG binds to the NtcA regulatory domain. This domain is responsible for the NtcA dimeric nature (Figure 6A) (Llácer et al., 2010; Zhao et al., 2010a). The regulatory domain of NtcA is highly similar to the corresponding domain of CRP (Llácer et al., 2010). The main differences reflect the changes in the characteristics of the site for the allosteric effector that enable the accommodation of 2OG instead of cAMP. Each 2OG molecule interacts in NtcA with the two (one per subunit) long interfacial helices that form the molecular backbone, crossing the molecule in its longer dimension, linking in each subunit both domains (Figures 6A,C) (Llácer et al., 2010; Zhao et al., 2010a). 2OG interactions with both interfacial helices favor a twist of one helix relative to the other, dragging the DNA binding domains and helices to apparently appropriate positions and interhelical distance for binding in two adjacent turns of the major groove of DNA where the NtcA box should be found (Figures 6A,B, and inset therein), although the experimental structure of DNA-bound NtcA should be determined to corroborate this proposals.

Although NtcA and CRP boxes are quite similar, plasmon resonance experiments (Forcada-Nadal et al., 2014) revealed that CRP exhibits complete selectivity and specificity for the CRP box, with absolute dependency on the presence of cAMP. In contrast, NtcA had less strict selectivity, since it still could bind to its promoters in the absence of 2OG, although with reduced affinity, and it could also bind to the the CRP promoter tested. Nevertheless, it is unlikely that NtcA could bind *in vivo* to the CRP boxes of cyanobacteria in those species where CRP is also present, given the much higher affinities for the CRP sites of cyanobacterial CRP and the relative concentrations in the cell of both transcriptional regulators (Forcada-Nadal et al., 2014).

While the structures of 2OG-bound NtcA of *S. elongatus* (Llácer et al., 2010) and of *Anabaena* (Zhao et al., 2010a) are virtually identical, the reported structures of “inactive” NtcA of *Anabaena* without 2OG (Zhao et al., 2010a) and of *S. elongatus* (Llácer et al., 2010) differed quite importantly in the positioning of the DNA binding domains (Figure 1, “Inactive forms” under “NtcA”), although in both cases the DNA binding helices were misplaced for properly accommodating the NtcA box of DNA, raising the question of whether these structural differences are species-specific or whether “inactive” NtcA can be in a multiplicity of conformations.

## PipX as an NtcA co-activator

Soon after PipX was found to interact with NtcA (Espinosa et al., 2006), it was also shown to activate *in vivo* transcription of NtcA-dependent promoters under conditions of low nitrogen availability (Espinosa et al., 2006, 2007). Direct binding studies with the isolated molecules proved that PipX binding to NtcA requires 2OG (Espinosa et al., 2006). Nevertheless, as PipX was not totally essential for transcription of NtcA-dependent promoters (Espinosa et al., 2007; Camargo et al., 2014), it was concluded (1) that PipX was a coactivator of 2OG-activated NtcA-mediated transcription (Espinosa et al., 2006; Llácer et al., 2010); and (2) that the degree of activation by PipX depended on the specific NtcA-dependent promoter (Espinosa et al., 2007; Forcada-Nadal et al., 2014). Detailed plasmon resonance studies (Forcada-Nadal et al., 2014) using sensorchip-bound DNA confirmed for three *Synechocystis* promoters that PipX binding to promoter-bound NtcA has an absolute requirement for 2OG, since no PipX binding was observed when NtcA was bound to the DNA in absence of 2OG. In these studies PipX increased about one order of magnitude the apparent affinity of NtcA for 2OG. In other *in vitro* experiments with four NtcA-dependent promoters of *Anabaena* sp. PCC 7120, PipX was also found to positively affect NtcA binding to its DNA sites (Camargo et al., 2014). The induction by PipX of increased NtcA affinity for 2OG and for its promoters could account for the PipX-triggered enhancement of NtcA-dependent transcription.

**Figure 7:**
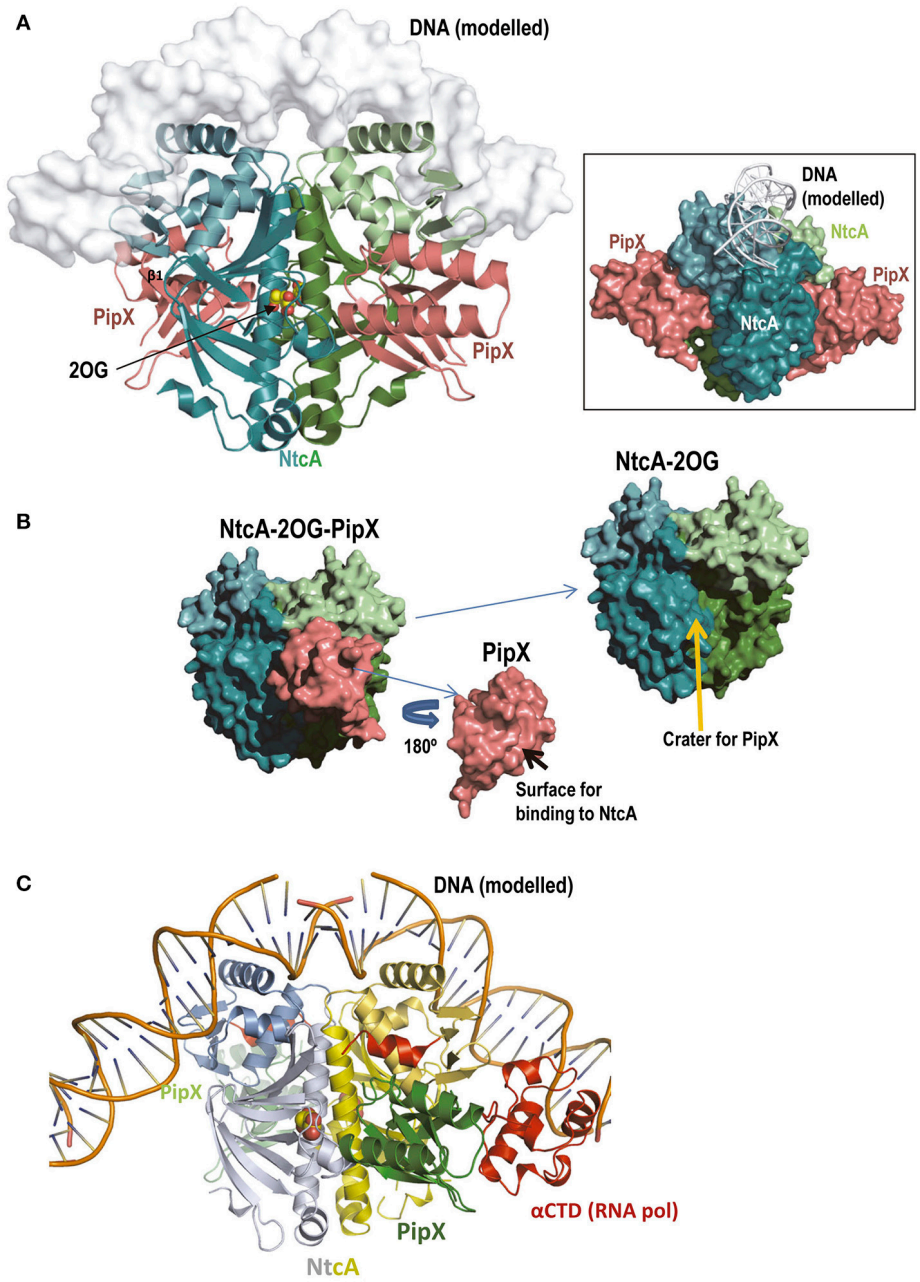
The NtcA-PipX complex. **(A)** Structure of the complex of *S. elongatus* NtcA and PipX, with DNA modeled in semi-transparent surface representation (Llácer et al., 2010). The projection of NtcA differs somewhat from that in Figure 6A to allow visualization of both PipX molecules, illustrating the fact that the Tudor-like domain is the part of PipX that binds to NtcA. Note that the two PipX molecules (the asymmetric unit contained an entire complex with two PipX molecules, PDB file 2XKO) are in the “flexed” conformation, that the flexed helices protrude away from the NtcA molecule and that they do not contact the modeled bound DNA (main figure and inset). The *inset* represents the same complex in a different orientation (viewed approximately along the DNA) and with all elements in surface representation except the DNA (shown in cartoon) to better visualize the protrusion in large part due to the flexed helices of both PipX molecules. **(B)** Deconstruction of the NtcA-PipX complex to show in surface representation the crater at the NtcA surface where PipX binds, and the surface of Pip X used in this binding. **(C)** A model based on CRP (Llácer et al., 2010) for the complex of the NtcAPipX complex with DNA and with the C-terminal domain of the α-subunit of RNA polymerase (αCTD), to show that the C-terminal helices of PipX could reach this part of the polymerase. In this figure the C-terminal helix of NtcA is colored red because it has no counterpart in CRP and is involved in the interactions with PipX.

The crystal structure of the NtcA-PipX complex of *S. elongatus* (Figure 7) (Llácer et al., 2010) corresponded to one “active” NtcA dimer with one molecule of each 2OG and PipX bound to each subunit. PipX is inserted via its Tudor-like domain (Figure 7A), filling a crater-like cavity formed over each NtcA subunit (Figure 7B) largely over one regulatory domain, being limited between the DNA binding domain and the long interfacial helix of the same subunit, and the regulatory domain of the other subunit. PipX extensively interacts with the entire crater, with nearly 1200 Å^2^ of NtcA surface covered by each PipX molecule, of which 65% belongs to one subunit (40%, 15% and 10% belonging to the DNA-binding domain, the interfacial helix and the regulatory domain, respectively) and 35% belongs to the regulatory domain (including the interfacial helix) of the other subunit, gluing together the elements of half of the NtcA dimer in its active conformation, stabilizing this conformation (Llácer et al., 2010). This conformation is the one that binds 2OG and that should have high affinity for the DNA, thus explaining the requirement of 2OG for PipX binding and the increased affinities of NtcA for 2OG and DNA when PipX was bound to NtcA (Forcada-Nadal et al., 2014). Since a similar crater-like cavity exists in other transcription factors of the CRP family including CRP (see for example McKay and Steitz, 1981 or Weber and Steitz, 1987) it would be conceivable that PipX-mimicking proteins could exist for these other transcriptional regulators of the CRP family, although PipX cannot do such a role since it does not bind to CRP (Forcada-Nadal et al., 2014). Furthermore, a large set of highly specific contacts (Llácer et al., 2010) ensure the specificity of the binding of PipX to NtcA.

The elements of the Tudor-like domain that interact with NtcA are largely the same that interact with the flat surface of the hemispheric body of P_II_ (many of them mediated by the upper layer of the Tudor-like β-sandwich, particularly strands β1 and β2), predicting total incompatibility for the simultaneous involvement of PipX in the NtcA and P_II_ complexes (Llácer et al., 2010). While the Tudor-like domain monopolizes the contacts of PipX with NtcA, the C-terminal helices of PipX do not participate in these contacts and remain flexed, as in isolated PipX (Forcada-Nadal et al., 2017), protruding away from the complex (Figure 7A). The coactivation functions of PipX for NtcA-mediated transcription could also involve these helices. However, *in vitro* experiments (Llácer et al., 2010; Camargo et al., 2014) and modeling (based on CRP) of DNA binding by the NtcA-PipX complex (Figure 7A and inset therein) (Llácer et al., 2010) did not support the idea of PipX binding to DNA. Alternatively, these helices could interact with RNA polymerase, particularly given the location, in the homologous CRP-DNA complex, of the binding site for the α-subunit of the C-terminal domain (αCTD) of RNA polymerase (Figure 7C; and discussed in Llácer et al., 2010). Further structures of P_II_-PipX bound to DNA alone or with at least some elements of the polymerase are needed to clarify these issues.

## The PipX regulatory network in quantitative perspective

The gene encoding P_II_ could not be deleted in *S. elongatus* unless the *pipX* gene was previously inactivated (Espinosa et al., 2009). Further studies led to the conclusion that decreasing the P_II_/PipX ratio results in lethality in *S. elongatus*, indicating that PipX sequestration into P_II_-PipX complexes is crucial for survival and implicating both proteins in the regulation of essential processes (Espinosa et al., 2010, 2018; Laichoubi et al., 2012). The ability of P_II_ to prevent the toxicity of PipX suggests that P_II_ acts as a PipX sink even under conditions in which the affinity for NtcA would be highest, supporting the idea that not all PipX effects are related to its role as NtcA co-activator. Mutational studies (Espinosa et al., 2009, 2010) and massive transcriptomic studies of *S. elongatus* mutants centered on PipX (Espinosa et al., 2014) also support the multifunctionality of PipX, stressing the need for additional studies, including the determination of PlmA functions and the search for further potential PipX-interacting proteins (Figure 1, broken yellow arrow).

**Figure 8:**
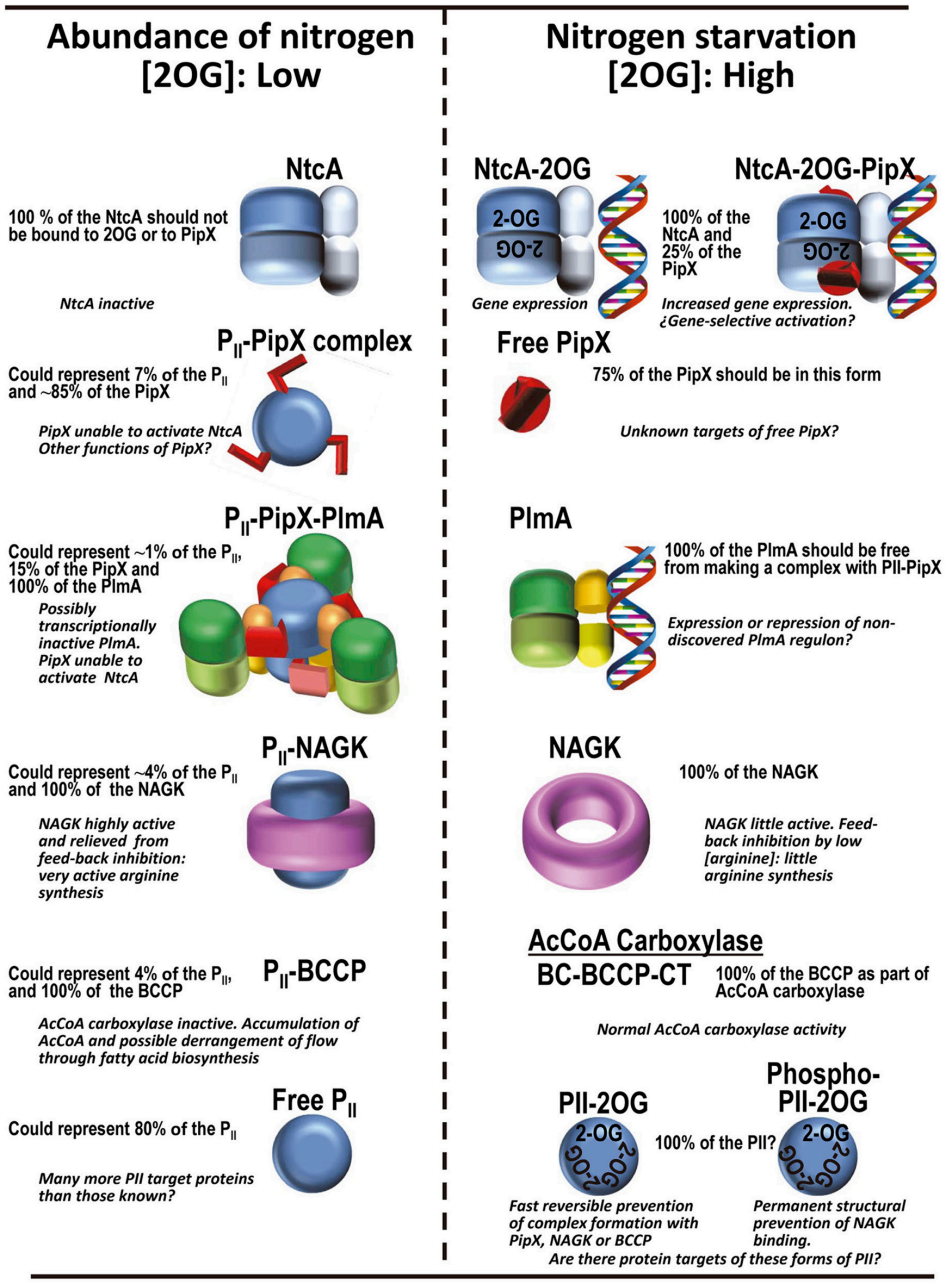
Protein complexes of the P_II_ regulatory system in *S. elongatus* according to availability of ammonium in the cell, and their corresponding functional consequences. The frequencies of the different chains in the various forms are based on the levels of the proteins in the cell found in proteomic studies (Table 1). The P_II_ trimer has been colored blue, PipX and its C-terminal helices are red, PlmA dimers have their DNA binding domains yellow or orange and their dimerization domains in two hues of green, NAGK is shown as a purple crown, and the regulatory and DNA binding domains of NtcA are given dark and light shades of blue, respectively.

Massive proteomic studies (Guerreiro et al., 2014) have estimated the number of chains of each protein of the P_II_-PipX network (Figure 1) in *S. elongatus* cells. The values obtained (Table 1) are corroborated by those obtained in focused western blot studies for some of these macromolecules (Table 1) (Labella et al., 2016, 2017). These quantitative data give an opportunity to evaluate the possible frequency of the different complexes and macromolecules of the P_II_-PipX-NtcA network (Figure 1) in one or another form (schematized in Figure 8). Of all the proteins mentioned here until now, P_II_ is by far the most abundant in terms of polypeptide chains (Table 1). In comparison, the sum of all the chains of other known P_II_-binding proteins represents no more than 20% of the P_II_ chains. Among these molecules is PipX, which only represents ~10% of all the P_II_ chains. This indicates that P_II_ has the potential to sequester all the PipX that is present in the cell (Figure 8). In turn, PlmA could be fully trapped in the P_II_-PipX-PlmA complex if this complex has the 1:1:1 stoichiometry proposed for it (Figure 1) (Labella et al., 2016), since the number of PlmA chains only represent about 10% of the number of PipX chains. Thus, about 10 and 1% of the P_II_ trimer could be as the respective P_II_-PipX and P_II_-PipX-PlmA complexes under nitrogen abundance conditions. In contrast, with nitrogen starvation all of the NtcA could be bound to PipX (Figure 8), given the ~five-fold excess of PipX chains over NtcA chains (Table 1). Thus, assuming that under conditions at which 2OG and ATP reach high levels P_II_ is totally unable to bind PipX, ~80% of the PipX molecules could be free to interact with additional protein partners.

**Table 1:** Levels of macromolecular players of the P_II_-NAGK-PipX-NtcA system in *S. elongatus*.

**Protein**	**Protein chains****^a^**		

	**N°copies/cell**	**As percentage relative to P**${}_{\text{II}}^{\text{b}}$	
P_II_	60,000	100	(100)
PipX	4,560	~8	(7)
NAGK	2,150	~4
BCCP	2,410	4
NtcA	1,000	~2
PlmA	400	~1	(1)
PipY^c^	1,275	2	(1.6)
PamA^c^ ^d^	36	< 0.1

*When given as percentages, the data are relative to the number of P_II_ chains (given the value of 100)*.

a *Data from massive proteomic study (Guerreiro et al., 2014). Percentages within parentheses are data based on immunoquantification in Western blots (Labella et al., 2016, 2017)*.

^b^Rounded to the closest integer or to the closest first decimal figure.

c *Given for reference, since there is no evidence of physical interaction with any of the other proteins*.

d *The physical interaction of this putative channel with P_II_ was found in Synechocystis sp. PCC6803, but the findings were not replicated in S. elongatus with the homologous product of gene Synpcc7942_0610*.

These inferences are consistent with the K_D_ values for the P_II_-NAGK (Llácer et al., 2007) and P_II_-PipX complexes in the absence of 2OG (Llácer et al., 2010) and for the PipX-NtcA complex at high 2OG and ATP (Forcada-Nadal et al., 2014) (~0.08, 7 and 0.09 μM, respectively). For the estimated cellular levels of the different components (Table 1), assuming a cell volume of 10^−12^ ml, virtually all the NAGK and ~95% of the PipX could be P_II_-bound in the absence of 2OG, and ~98% of the NtcA could be PipX-complexed in the presence of 2OG. However, the impacts of varying concentrations of 2OG on the disassembly or assembly of the complexes most likely differ for the various complexes. For example, a two-order of magnitude increase in the K_D_ value for the P_II_-NAGK complex due to 2OG binding might have much less impact (a 7% decrease in the amount of NAGK bound to P_II_ would be estimated from the mere total protein levels and K_D_ value) than a two-order of magnitude increase in the K_D_ for the P_II_-PipX complex (an 80% decrease in the amount of PipX bound estimated similarly). These estimations are very crude, since they do not take in consideration that in *S. elongatus* P_II_ phosphorylation prevents NAGK binding (Heinrich et al., 2004), and that this phosphorylation is greatly influenced by the abundance of ammonia (Forchhammer and Tandeau de Marsac, 1994). Furthermore, we have not considered in these estimates the influence of the ATP concentrations, recently shown to decrease *in vivo* in *S. elongatus* upon nitrogen starvation (Doello et al., 2018). Therefore, the situation is much more complex than would be expected from the mere consideration of the abundances of the different proteins and of the K_D_ values for the non-phosphorylated form of P_II_. Nevertheless, it appears desirable to estimate the influence of different 2OG levels on K_D_ values as an important element to take into consideration in future attempts to model the concentrations of the different complexes of P_II_ and PipX under intermediate conditions of nitrogen richness.

## A novel network member functionally related to PipX

Recently, a novel protein has been identified as belonging to the PipX regulatory network (Figure 1, yellow 3D arrow projecting upwards from the plane of the network). In this case no direct protein-protein interaction with PipX has been shown (Labella et al., 2017). This protein (PipY), is the product of the downstream gene in the bicistronic *pipXY* operon. The regulatory influence of PipX on PipY was originally detected in functional, gene expression and mutational studies (Labella et al., 2017). More recently it has been concluded (Cantos et al., 2018) that PipX enhances *pipY* expression in *cis*, preventing operon polarity, a function that might implicate additional interactions of PipX with the transcription and translation machineries, by analogy with the action of NusG paralogues, which are proteins bearing, as PipX, Tudor-like domains. It has been proposed (Cantos et al., 2018) that the *cis*-acting function of PipX might be a sophisticated strategy for keeping the appropriate PipX-PipY stoichiometry.

PipY is an intriguing pyridoxal phosphate-containing protein that is folded as a modified TIM-barrel (Figure 1). The PipY structure (Tremiño et al., 2017) gives full structural backing to unsuccessful experimental attempts to show enzymatic activity of PipY and its orthologs (Ito et al., 2013). Because of these negative findings, and given the pleotropic effects of the inactivation of the PipY orthologs in microorganisms and humans, it has been concluded that these proteins have as yet unclarified roles in PLP homeostasis (Ito et al., 2013; Darin et al., 2016; Prunetti et al., 2016; Labella et al., 2017; Plecko et al., 2017). Interestingly, these proteins are widespread across phyla (Prunetti et al., 2016) and the deficiency of the human ortholog causes vitamin B_6_-dependent epilepsy (Darin et al., 2016; Plecko et al., 2017; Tremiño et al., 2018), providing an excellent example that investigations of cyanobacterial regulatory systems like the one summarized here can have far-reaching consequences spanning up to the realm of human and animal pathology. If any lesson can be inferred from all of the above, is that the investigation on P_II_ and particularly on PipX proteins require further efforts.

## Final remarks

The rich P_II_ regulatory network summarized in Figure 1 of this review even for a unicellular microorganism with a single type of P_II_ protein, attests the importance of P_II_ and of its regulatory processes. This importance, possibly underrecognized until now, is highlighted, for example, by the very wide distribution of P_II_ proteins among microorganisms and plants. Furthermore, in the many organisms with several genes for P_II_ proteins the levels of complexity expected from P_II_ regulatory networks may be much greater than the one presented here. Each one of these paralogous P_II_ proteins may command a regulatory network, and it would be unlikely that these networks would not be interconnected into a large meshwork that will require the instruments of systems biology to be fully understood.

PipX also deserves deeper attention than received until now. Massive transcriptomics studies (Espinosa et al., 2014) have ascribed to this protein a paramount regulatory role in *S. elongatus*. For full understanding of this role further searches for PipX-interacting or functional partners like PipY appear desirable, with detailed investigation of the molecular mechanisms of the physical or the functional interactions. PlmA merits particular attention to try to characterize the roles of the P_II_-PipX-PlmA complex. PipY and its orthologs deserve similar attention, to try to define molecularly their PLP homeostatic functions, a need that is made more urgent by the role in pathology of the human ortholog of PipY. In addition to all of this, the structural evidence reviewed here makes conceivable that adaptor proteins capable of stabilizing active conformations of other transcriptional regulators of the CRP family could exist outside cyanobacteria, mimicking the PipX role. In summary, there are many important questions to be addressed arising from the field reviewed here, some within cyanobacteria, but others concerning whether the mechanisms and complexes exemplified here could have a parallel in other bacterial or even plant species. Clearly more investigations on P_II_ and its partners in other phylogenetic groups using the approaches and experimental instruments used to uncover the cyanobacterial P_II_ regulatory network would appear highly desirable.

## Author contributions

AF-N, JLL, AC, CM-M, and VR reviewed the literature and their own previous work and contributed to the discussions for writing this review. The main writer was VR, but all the authors contributed to the writing of the manuscript. AF-N and CM-M prepared the figures, with key inputs from VR.

### Conflict of interest statement

The authors declare that the research was conducted in the absence of any commercial or financial relationships that could be construed as a potential conflict of interest. The reviewer JAH declared a shared affiliation, with no collaboration, with several of the authors, AF-N, JL, CM-M, and VR to the handling editor at time of review.

## Abbreviations

P_II_: a homotrimeric signaling protein
GlnK, GlnK3 and GlnB: different paralogous forms of P_II_ proteins
GlnD: the bifunctional enzyme that uridylylates and deuridylylates GlnB in *E. coli*
GS: glutamine synthetase
AmtB and Amt: homologous trimeric bacterial transporters of ammonia
PamA: putative channel of unknown function that is encoded by *sll0985* of *Synechocystis* sp. PCC 6803
NtcA and CRP, homologous homodimeric transcription factors of cyanobacteria and of *E. coli*: respectively
the imperfectly palindromic target DNA sequences to which they bind specifically are called NtcA box and CRP box: respectively
PlmA: putative homodimeric transcription factor of the GntR family that is found in cyanobacteria
P_I_ or ATase: bifunctional enzyme that adenylylates and deadenylylates glutamine synthetase in *E. coli* and other enterobacteria
NAGK: N-acetyl-L-glutamate kinase
NAG: N-acetyl-L-glutamate
PipX: a small monomeric protein of cyanobacteria that can interact with P_II_ and with NtcA
FRET, fluorescence resonance energy transfer (also known as Förster resonance energy transfer): a phenomenon in which a fluorophore emits light of its characteristic frequency when a nearby different absorbing group is excited by light
the FRET signal decreases with the 6th power of the distance between the absorbing and emitting groups
2OG, 2-oxoglutarate: also called α-ketoglutarate
AcCoA carboxylase: acetyl coenzyme A carboxylase
BCCP, biotin carboxyl carrier protein: the protein subunit that hosts the covalently bound biotin in many bacterial AcCoA carboxylases
PLP: pyridoxal phosphate
PipY, product of the gene that in *S. elongatus* is the next downstream of pipX: forming a bicistronic operon with it
it is a PLP-containing protein.
